# Clinical and molecular characterization of patients with classic 3β-hydroxysteroid dehydrogenase deficiency

**DOI:** 10.1186/1687-9856-2015-S1-P43

**Published:** 2015-04-28

**Authors:** Pattara Wiromrat, Kewalee Unajak, Viral Shah, Taninee Sahakitrungruang

**Affiliations:** 1 Chulalongkorn University, Bangkok, Thailand; 2 Chiang Mai University, Chiang Mai, Thailand; 3 Government medical college, Bhavnagar, Gujarat, India

## Background

3β-hydroxysteroid dehydrogenase type 2 (3βHSD2) is the key enzyme converting Δ5-steroids to Δ4-ketosteroids in adrenal and gonadal steroidogenesis. Severe loss-of-function mutations of HSD3B2 gene encoding for this enzyme cause the rare form of congenital adrenal hyperplasia, “3βHSD deficiency”. Affected individuals have salt losing, adrenal insufficiency and ambiguous genitalia in both sexes. Patients with 3βHSD deficiency may have elevated 17α-hydroxyprogesterone (17OHP) levels due to normal peripheral type 1, 3βHSD.

## Aims

To describe two unrelated patients with 3β-hydroxysteroid dehydrogenase deficiency and perform mutation analysis of the HSD3B2 gene.

## Patients and Methods

Patient 1 (Thai) and 2 (Indian) are 46,XY male newborns with ambiguous genitalia (micropenis, penoscrotal hypospadias) who developed salt-losing since early infancy. They were stabilized with normal saline resuscitation and high dose hydrocortisone replacement. Patient 2 was initially misdiagnosed as 21-hydroxylase deficiency due to elevated 17OHP until he was referred for genitoplasty at the age of 2.5 years and the patient were re-evaluated. The ACTH tests revealed low cortisol response, moderately elevated 17OHP, elevated ∆^5^/∆^4^ steroids, suggestive of blockage at the level of enzyme 3βHSD. Patients’ leukocyte genomic DNA was extracted and the entire coding regions of the HSD3B2 gene were assessed by polymerase chain reaction (PCR) and sequencing analysis.

## Results

Patient 1 was homozygous for T259M (c.776C>T) mutation in the HSD3B2 gene. Patient 2 was homozygous for the novel nonsense mutation Y180X (c.540C>A) and his parents were heterozygous carrier.

## Conclusion

We report the mutations of HSD3B2 gene, T259M and Y180X (novel) responsible for classic 3βHSD deficiency. The clinical and hormonal phenotypes can be complicated in this disorder. These cases emphasize the importance of confirming the specific enzyme deficiency with molecular genetic analysis.

Written informed consent was obtained from the patient for publication of this abstract and any accompanying images. A copy of the written consent is available for review by the Editor of this journal.

